# Hepatocellular recurrence after orthotopic liver transplantation: Is combination of α-fetoprotein and glypican-3 a reliable marker? Hepatocellular recurrence after orthotopic liver trasplantation

**Published:** 2011-03-01

**Authors:** Mohammad Hashemi, Saeid Ghavami

**Affiliations:** 1 Department of Clinical Biochemistry, School of Medicine, Zahedan University of Medical Sciences, Zahedan, IR Iran; 2 Manitoba Institute of Child Health, Department of Physiology, University of Manitoba, Winnipeg, Canada

**Keywords:** Liver transplantation, Alpha fetoproteins, Glypican-3

Wang, et al. have provided new and novel evidence about the importance of using α-fetoprotein (ALP) and glypican-3 (GPC-3) mRNA detection in peripheral blood of the patients with hepatocellular carcinoma (HCC) recurrence after ortothopic liver transplantation (OLT) [1]. HCC keeps on as major universal health apprehension ensuing in more than 1 million caner associated deaths annually [2][3]. These results are frankly attributable to a commonly pitiable prognosis for patients with HCC, with few attaining 5-year survival [3]. Although the incidence of HCC in North America has been comparatively low, a stable augmentation has taken place since the 1980s, while the prevalence of most other cancers has turned down [4]. Because of the comparative resistance of HCC to systemic chemotherapy, pioneering treatments such as transarterial chemoembolization and yttrium 90-labeled radiotherapy has been extended to improve disease manage.

HCC often happens in the setting of cirrhosis, total hepatectomy with OLT affords one of the only prospective cures for both diseases so at present liver transplantation has been established as a cure of patients with HCC and associated cirrhosis. In spite of this, the return of HCC after liver transplantation remains an unanswered concern. Mazzaferro et al. proved that a stringent assortment of HCC patients, focusing on tumor-staging before transplantation, could facilitate an acceptable tumor-free survival. They also found poor outcome among patients whose tumors did not descend within the criteria (i.e., the "Milano criteria") [5]. In general, immunosuppressed organ allograft recipients are at increased jeopardy of developing a number of malignancies. Vivarelli et al. [6] confirmed that the amount of immunosuppressant agents (cyclosporine) administered was associated with HCC recurrence after liver transplantation. Recent studies showed that the risk of HCC recurrence subsequent liver transplantation is larger with antibody treatment than with other classes of immunosuppressive agents [7]. Several researches have been performed to find a reliable tumor marker for HCC and HCC recurrence after OLT. During the past 50 years, AFP has been used as a serum tumor marker for HCC, but its function as a probable predictive factor in OLT for HCC has not been established. AFP is a major transport protein in the fetus [8] and being secreted in only about 70% of HCC cases, so both false-negative and false-positive rates are elevated when considering AFP as the serological marker for the detection of HCC [9]. In general, patients have an initially high serum AFP level when HCC is diagnosed. Serum AFP level is also considered an effective tumor marker in the surveillance of HCC recurrence [10]. It is expected that recurrent HCC to have the identical bioactivity as the primary tumor and to exude high levels of AFP, making AFP an apparently reliable choice. Regular checks of serum AFP levels in patients who originally had high AFP levels to recognize HCC recurrence is suggested as a practice guideline by the National Comprehensive Cancer Network (NCCN) version 1, 2009.

The whole AFP can be alienated into three different sub-fractions, AFP-L1, AFP-L2, and AFP-L3, based on its reactivity to LCA on affinity electrophoresis. AFP-L1 does not react with LCA. It is increased in chronic hepatitis and liver cirrhosis and comprises a bulk fraction of the total AFP in non-malignant liver diseases. AFP-L2 is mostly derived from yolk sac tumors and could also be detected in maternal serum during pregnancy. AFP-L3 is the LCA-bound fraction of AFP. It has been reported that malignant liver cells produce AFP-L3, still when HCC is at its near the beginning stages [11]. AFP-L3 has been established to be a marker for HCC with a high specificity of 95%. AFP-L3-secreting hepatocytes have an increased tendency for rapid growth, early attack, and intrahepatic metastasis, thus leading to a poorer prognosis in affected individuals [12][13]. AFP and AFP-L3 levels decline to normal levels with effective therapy, and then rising levels suggest disease progression or recurrence. Therefore combination of total AFP and AFP-L3 could be a very reliable biomarker for HCC and HCC recurrence in OLT.

GPC-3 (also called DGSX, GTR2-2, MXR7, OCI-5, SDYS, SGB, SGBS, and SGBS1), a cell exterior protein, is extremely expressed in HCC and some other human cancers including melanoma [14]. The GPC3 gene encodes a 70-kDa-precursor core protein, which can be sliced by furin to make a 40-kDa amino (N) terminal protein and a 30-kDa membrane-bound carboxyl (C) terminal protein, which has two heparan sulphate (HS) glycan chains [15]. The GPC3 protein is attached to the cell membrane by a glycosyl-phosphatidylinositol (GPI) anchor [16]. GPC-3 is not expressed in hepatocytes of healthy subjects and patients with non-malignant hepatopathy, and can be detected in about 50% of HCC patients and 33% of HCC patients seronegative for AFP. The specificity of GPC-3 could be considered 100% according to some previous research [17]. Some clinical studies have indicated that the simultaneous determination of GPC-3 and AFP could significantly increase the sensitivity in HCC detection, without a reduction in the specificity [18]. The clinical significance of changes in tumor markers after therapy has been investigated for various solid tumors, and these changes are closely correlated with tumor response. For HCC, early studies have shown that AFP levels decrease rapidly after complete surgical resection and increase with tumor recurrence, suggesting that AFP response could potentially predict tumor response [19]. A few recent studies highlighted the potential role of serial AFP screening in the evaluation of treatment response, with the supposition that AFP is indicative of tumor growth and activity [20][21]. Regarding the new research combination of total AFP, AFP-L3, and GPC-3 could be a good marker for HCC recurrence after OLT. Therefore Wang et al paper in this volume pointed out a very good beginning to monitor HCC patients who have under-gone the OLT and needs monitoring for tumor recurrence.
